# Allometric scaling for chemical restraint in greater Rheas (*Rhea americana*) with Tiletamine and Zolazepam

**DOI:** 10.1186/1746-6148-10-66

**Published:** 2014-03-13

**Authors:** Bruno Carneiro Pinheiro, Dayanne A Silva Dantas Lima, Wagner C Lima, Ana Maria Quessada, Marcelo C Rodrigues

**Affiliations:** 1 Department of Veterinary Clinic and Surgery, Campus do Socopo, Federal University of Piauí, Desembargador Robert Wall de Carvalho Street, n. 949, apt. 102, Ininga, Teresina, Brazil; 2 Federal University of Piauí, Campus Cinobelina Elvas, 64900-000 Bom Jesus, Brazil; 3 Universidade Paranaense, UNIPAR, 87502-970 Umuarama, Paraná, Brazil

**Keywords:** Allometric scaling, Chemical restraint, Greater rhea, Rhea americana, Tiletamine, Zolazepam

## Abstract

**Background:**

Chemical restraint is of great importance in the clinical practice of wildlife animals. In such, interspecific allometric scaling proposes pharmacological doses to a wide range of species, based on previously known doses for domestic animals and the target animal’s body mass. The objective was to compare chemical restraint responses in the greater rhea (*Rhea americana*) with conventional doses of tiletamine/zolazepam, found in the literature for the species, and with doses calculated through interspecific allometric scaling extrapolation. From the Federal University of Piauí, six adult greater rheas (*Rhea americana*), three males and three females, were randomly selected to be subjects in this research. All six animals were submitted to two chemical restraint protocols with tiletamine and zolazepam, per intramuscular injection in the hind limb. The first protocol was composed of doses found on the literature for the species, while the second protocol used doses calculated by interspecific allometric scaling, with the domestic dog as model animal. Heart and respiratory rates, body temperature, eyelid reflex, digital pinch and metatarsal reflex were registered along with latency and ambulation times.

**Results:**

The use of interspecific allometric scaling for chemical restraint with the combination tiletamine and zolazepam showed satisfying results, with great similarity to results obtained with conventional doses in Greater rheas.

**Conclusions:**

Literature on chemical restraint and use of tiletamine and zolazepam in rheas is scarce. Chemical restraint is of extreme importance on these animals, due to their aggressive nature and low level of domesticity. This research may further establish the interspecific allometric scaling method as a viable tool for the veterinary physician in formulating anesthetic and chemical restraint protocols for wildlife animals.

## Background

Physical restraint is a recurring problem in wildlife clinic, by submitting these animals to different levels of stress. Their lower degree of domesticity makes them more susceptible to damaging effects of tension, and minimize them is of great interest to the veterinary [1].

The greater rhea (*Rhea americana*) is a ratite bird, largely distributed in northeast and southeast of Brazil, as well as Bolivia, Uruguay, Paraguay and north of Argentina. Since the 90s decade, it is considered a near threatened species, due to hunting for skin and meat and destruction of its natural habitat. The greater rhea and other species of the Rheidae family are animals of livestock interest, especially in Argentina and Uruguay [2].

In livestock, management and transportation of animals can be deemed indispensable. Studies showed a 40 fold increase in serum levels of corticosteroids in greater rheas after a transportation of only 30 minutes, compared to animals who were not submitted to transportation [3], while behavioral pattern changes in those same animals after transportation were also reported, with increase in vigilance and decreasing in feeding times [4].

As such, chemical restraint can become essential in managing these and other wild animals, even for simple maneuvers as clinical exams, biological samples collection and management techniques like capture and transportation. It provides several advantages, neutralizing possible aggressive behaviors, providing safety for all evolved and can avoid traumatic memories for the animal through amnesia [5,6]. Physical restraining is mostly inadequate in greater rheas, due to their size, aggressive nature and powerful hind limbs, leading to risks of self-trauma and to surrounding personnel [7].

However, there is considerable shortage of studies for dosing anesthetic and tranquilizing drugs in wildlife animals [8], especially rheiforms [7], with estimated doses from domestic animals conventionally used. In view of their anatomical and physiological differences, such estimates can be deemed crude [9]. Furthermore, pharmacological studies through real-time plasmatic dosing are impractical or outright impossible in certain species [10].

Interspecific allometric scaling can provide a tool for calculating drug doses for animals that lack established doses, based on established doses for domestic animals or humans. It can be a versatile method for the veterinary physician, adapting to a wide range of exotic and wildlife species and taxa [9]. The calculation is based on the energy consumption of each animal, proportional to body mass and adjusted according to the taxonomical group, showing efficiency if particular and individual characteristics of each animal order are respected. Through that method, it is possible to mathematically extrapolate drug dosage to a target animal, based of doses from a model animal (domestic animal or human) [8].

Dissociative anesthetics are among the most used drugs for anesthesia, tranquilization and chemical restraint of wildlife animals. These drugs provide easy application and can be considered safe, due to minimum cardiac depressive effects [11].

As such, tiletamine has its anesthetic activity through interaction with NMDA (N-Metil-D-aspartate) receptors, preventing it’s activation by stimulant neurotransmitter glutamate in the central nervous system. This activity would mediate the positive nervous cardiovascular effects of tiletamine [11,12], resulting in an increase in heart rate, blood pressure and body temperature upon application [13,14]. The zolazepam is a benzodiazepine commonly associated in this drug, on a 1:1 ratio, providing greater muscle relaxation and smoother recovery from anaesthesia [11,12].

The objective of this research study is to compare the chemical restraint response of greater rheas with tiletamine/zolazepam, in two protocols: conventional doses, found in the literature for the species, and allometric scaled doses of tiletamine and zolazepam, using the domestic dog as model animal. As such, it bring forward the role of interspecific allometric scaling as a useful tool for the veterinary physician in the fields of clinic, management and anesthesiology of exotic and wildlife animals.

## Methods

Six adult, male greater rheas (*Rhea Americana*), median weight 25.2 ± 5.6 kg, were randomly selected from the University of Piauí, city of Teresina, provided by the Research and Preservation of Wildlife Animals Center (NEPPAS). All animals were young adults (although their exact ages were unknown) and were kept at NEPPAS for research purposes. The entire experiment took place at an open grassland at NEPPAS (5°02′S 42°46′W), where the animals already lived, and were removed from their site only during protocol application, being returned after a full recovery, always no more than two hours later. The city of Teresina, where the research took place, has an average temperature of 28 ± 11.5°C and an average photoperiod of 12 hours and 19 minutes per day [15].

The animals were individually marked by nylon bands in the tibiotarsal region of their right hind limbs. To avoid variation related to their circadian and biorhythm cycle, the entire experiment occurred on the early morning hours. The project was approved and followed all recommendations by the Animal Experiments Ethics Committee of the Federal University of Piauí under the protocol n. 018/11, as well as by the Chico Mendes Institute of Biodiversity Conservation (ICMBio), authorization number 28887–1.

Before inclusion in the experiment, each animal was physically examined and deemed healthy, with a good body score and no signs of lesions, anemia or parasitic diseases. All animals were submitted to two anesthetic protocols, labeled Conventional Protocol (CP) and Allometric Protocol (AP) using the association tiletamine and zolazepam by intramuscular injection in the right hind limb. The doses for CP were 12 mg/kg, [16,17] while for AP was calculated by interspecific allometric scaling [8,9] using the domestic dog (*Canis familiaris*) as model animal. All specimens received a minimum spacing of 7 to 10 days between each protocol application, as well as were weighted immediately before each protocol, subjected to fasting of 12 hours.

The interspecific allometric scaling protocols for AP were calculated as follows [8,18]:

1. Calculate the base metabolic rate (BMR) of the target-animal (Greater rheas, BMRt) and model-animal (dog, body mass 10 kg, BMRm) with the formulae BMR = kM^0.75^, where k is the taxonomic-specific allometric constant and M is the body mass.

2. Total dose for the target-animal, Qt, in mg, is provided by Qt = BMRt x Qm/BMRm, where Qm is the total dose (in mg) for the model-animal.

3. Dividing Qt by the target-animal’s body mass, we acquire the dose in mg/kg.

Noting that, in this case, total dose for the model-animal (Qm) is 100 mg (10 mg/kg [11]) and the taxonomic k constant is k = 70 for placental mammals (dog) and k = 78 for non-passeriform birds (greater rheas) [8].

The following physiological parameters were measured: heart rate (HR), using a clinical stethoscope, respiratory rate (RR), through observation of thoracic and abdominal movements of the animal, and body temperature (BT) in Celsius (°C) by a thermometer in the cloaca. Palpebral reflex (PR) and reflex of interdigital pinching (interdigital reflex, IR) and pinching above the digits (pedal reflex, PR) were tested, and latency time (time period between drug application and loss of postural reflex, or lateral decumbency), able immobilization time and recovery time (total time until spontaneous ambulation) were also measured. All parameters were registered in six moments, from immediately before drug application (M0) to intervals of 5 minutes for 30 total minutes after latency (moments M1 to M6). Other qualitative effects on animals were also observed.

All data was gathered and analyzed in an ANOVA and through the Student test t for significance of differences, with software OpenStat. Application of all protocols happened in the same place, with the same participant crew, further assisted by the caretaker who daily handles all the animals. During the entire recovery period, all animals were kept in an inflated bed, to provide comfort and safety, as an agitated recovery was anticipated, to avoid self-trauma [5].

## Results

As a whole, the association of tiletamine and zolazepam promoted anesthetic plane adequate for short and minimally invasive procedures, in both protocols, with satisfactory sedation and muscle relaxation. The doses on protocol AP were considerably different from those found on the literature for the species, from 8.5 mg/kg to 9.4 mg/kg, and can be seen on Table 1.

**Table 1:** **Tiletamine and zolazepam association doses in conventional protocol (CP) and allometric protocol (AP), according to body mass, for chemical restraint in greater rhea (*Rhea americana*)**

	**Animals**					
	**1**	**2**	**3**	**4**	**5**	**6**
**BM**	25 kg	24 kg	21 kg	30 kg	20 kg	28 kg
**CP**	12 mg/kg	12 mg/kg	12 mg/kg	12 mg/kg	12 mg/kg	12 mg/kg
**AP**	8.9 mg/kg	9 mg/kg	9.3 mg/kg	8.5 mg/kg	9.4 mg/kg	8.6 mg/kg

BM- body mass; CP - conventional protocol; AP – allometric.

The gathered data was homoscedastic, by Bartlett’s test, and normalized (Kolmogrov-Smirnov and Shapiro-Wilk tests) before the application of the ANOVA and t test.

Both groups showed no statistical difference in heart rates (HR) for all animals (p < .05), as both groups obtained equal median values for HR on moments M0 through M6. As such, in both protocols showed stable heart rate throughout the entire experimental period, as the HR observed on M0 = M1 = M2 … = M6 (p < .05) for CP and AP.

On respiratory rate (RR), the protocols showed significant difference (p < .05), with the AP providing a higher average then CP, 42.2 and 32.6 movements per minute, respectively. Evaluating each protocol separately, RR remained constant on the CP, while in the AP a higher RR was seen in moments M5 and M6 (25 and 30 minutes after latency), while M0 through M4 were constant (p < .05).

Body temperature (BT) showed a small, although significant, difference between protocols (p < .05), with CP and AP showing median temperatures of 41°C and 41.5°C respectively. There were no statistical differences between moments on each separate protocol. Detailed data on HR, RR and BT is available on Table 2.

**Table 2:** **Mean and standard deviations of heart rate, respiratory rate and body temperature in greater rheas (*Rhea americana*) chemically restrained with tiletamine/zolazepam, on conventional protocol and allometric protocol**

**Parameter**	**Protocol**	**Moments**						
		**M0**	**M1**	**M2**	**M3**	**M4**	**M5**	**M6**
**HR (bpm)**	**CP**	120.7 ± 5.3	140 ± 40	128.7 ± 26	125.7 ± 30.4	120.7 ± 38.3	110.7 ± 43	102.7 ± 41
	**AP**	124.7 ± 46.2	154 ± 40.5	142.7 ± 28,7	141.3 ± 33.9	122.3 ± 22.8	135 ± 23.4	119.7 ± 16.6
**RR (rpm)**	**CP**	26.33 ± 7.2	32.7 ± 10.6	30.3 ± 13,3	35.67 ± 11.8	34 ± 12.3	32.7 ± 9.3	36.7 ± 13.2
	**AP**	44.2 ± 23	32 ± 10.4	28.7 ± 14,6	38.7 ± 19.9	41.3 ± 18.7	53.3 ± 19.9	50 ± 13.8
**BT (°C)**	**CP**	40.5 ± 1	41.1 ± 0.5	41 ± 0,5	41 ± 0.4	41.1 ± 0.5	41.2 ± 0.4	41.3 ± 0.6
	**AP**	41.2 ± 0.5	41.3 ± 0.5	41.4 ± 0,3	41.6 ± 0.4	41.6 ± 0.5	41.6 ± 0.4	41.7 ± 0.5

HR - Heart rate, in beats per minute; RR - Respiratory rate, in cycles per minute; BT - Body temperature, in Celsius; CP - Conventional Protocol; AP - Allometric protocol.

Most animals maintained their monitored reflexes throughout the experiment, with only one animal, during allometric protocol, showing loss of reflexes for two consecutive moments (M2 and M3). Despite this indication of superficial analgesia, all animals on both protocols showed intense muscle relaxation for at least four consecutive experimental moments (20 minutes).

Latency times, measured from drug application to the animals’ complete lack of postural reflex, lateral decumbency and absence of resistance to handling, showed great stability among all animals from both protocols. On 10 of 12 total applications, latency times were between 3 and 4 minutes, with one case of 2 minutes (on CP), and one animal with latency of 5 minutes (on AP). Average times were 3 and 3.8 minutes for CP and AP, a difference considered statistically insignificant (p < .05).

Sedation time, however, showed great individual variance, on both groups, ranging from 35 to 67 minutes, as shown on greater detail on Table 3. Average times for this parameter were 51.5 minutes for conventional protocol and 46.2 minutes for the allometric protocol, values considered statistically equal (p < .05), as the individual variance basically evened out on both groups similarly. Total times for ambulation, on average, were 76.8 minutes for CP and 59.8 minutes for AP, also without statistical difference (p < .05).

**Table 3:** **Time, in minutes, for ventral decumbency in greater rheas (*Rhea americanCP*) chemically restrained with tiletamine and zolazepam, on conventional protocol (CP) and allometric protocol (AP), animals 1 through 6**

**Protocols**	**1**	**2**	**3**	**4**	**5**	**6**
**CP**	65 min	50 min	60 min	42 min	47 min	45 min
**AP**	67 min	56 min	35 min	40 min	40 min	29 min

CP - Conventional Protocol; AP - Allometric Protocol.

All plots showed evident agitated recovery, with predominantly vigorous paddling and neck movements, apparently trying to recover postural position. The animals were responsive to sound and touch stimuli, notably from moment M5 and M6 forward (from 25 minutes of latency onwards).

## Discussion

The drug application site is of importance on ratites and birds in general, due to the presence of the renal portal system, directing part of the venous blood from the hind limbs and general posterior region of the animal’s body through the kidneys before reaching the general circulation. This system may interfere directly with plasmatic concentration of drugs administered on this parts of the animal’s body [5]. However, ensuing data in ostriches (*Struthio camelus*) reports similar and satisfactory results with drugs administered on the fore limbs and hind limbs [16].

The maintenance of heart and respiratory rate stability inside normal parameters for the species was also observed in ostriches [5,19] using dissociative anesthetic agents coupled with benzodiazepines and α-2 agonists in conventional doses. In emus (*Dromaius novaehollandiae*), an increase in HR was reported using dissociative drugs, but this results were connected by the authors to the use of atropine as a pre-anesthetic drug [20]. By utilizing dissociative anesthetic agents through allometric scaled doses in domestic dogs [21], oncillas [22] (*Leopardus tigrinus*) and the giant anteater [23] (*Myrmecophaga tridactyla*), the same HR and RR stability was reported. The tiletamine has notable cardiovascular activity by central nervous system activation, while the zolazepam, as most benzodiazepines, have little effect on cardiorespiratory rates [24].

No studies referenced reported assessment of defense reflexes, like palpebral and pedal withdrawal reflexes. However, all reported sedation without deep analgesia, compatible with the use of tiletamine/zolazepam in chemical restraint or initial anaesthesia induction.

The use of dissociative anesthetic agents, coupled with xylazine, acepromazine and atropine, in conventional doses in ostriches and emus, resulted in observations very similar to this manuscript regarding muscle relaxation, sedation times and adequate recovery [5,20]. The use of xylazine specially is common coupled with dissociative anesthetics, usually inducing greater cardiac depression and muscle relaxation.

Bradycardia and bradypnea were reported in the use of tiletamine/zolazepam in small doses (5 mg/kg) associated with dexmedetomidine and thiafentanil, respectively a α-2 agonist and an opioid narcotic, in greater rheas [7]. These observations were most likely related to the drugs associated to tiletamine/zolazepam, instead of tiletamine/zolazepam itself. The authors call for the use of α-2 agonists or other drugs combined with tiletamine/zolazepam, citing better anesthesia and lower volume injected. However, cost of drug acquisition must be considered, as well as risks associated with the use of narcotic drugs.

Different results were obtained in induction time in ostriches using xylazine and acepromazine associated to tiletamine/zolazepam. Induction times, characterized by the authors as time from drug administration to loss of postural reflex, varied from 6 to 40 minutes, averaging around 20 minutes [5]. The methods, similar to the present manuscript, were hardly responsible for this difference. However, physiological characteristics, like body temperature and nutritional condition, may significantly affect metabolism of anesthetic drugs [6]. Specific differences between ostriches and rheas may also be considered. In emus, however, reported induction times with tiletamine/zolazepam/atropine were 40 to 60 seconds, and anesthesia time of 20 to 25 minutes, similar to this manuscript [20].

Agitated recovery after the use of tiletamine and zolazepam has been previously reported, highlighting sudden head and neck movements and with animals needing assistance to avoid self-trauma on the animals, similarly to what we report in this manuscript [5]. Such behavior cannot be considered completely abnormal in the use of dissociative agents [11], justifying its use associated with muscle relaxing drugs and tranquilizers, to avoid such effects [5,6,11]. The plasmatic half-life of zolazepam, however, may be smaller than that of tiletamine, explaining the excess of excitatory effects during the later stages of recovery [11].

A behavior of walking with open wings, with short-winded breathing and bristling feathers was observed in all animals a few minutes before and during initial stages of spontaneous ambulation. These reflexes may aim to regulate body temperature, coordinated mainly by the brain staff, beginning after partial recovery from sedation. Dissociative anesthetic agents partially or completely inhibit the nervous system’s control of main body functions, relating these observations with initial stages of recovery from anesthesia [6,12,14,19].

The interspecific allometric scaling method of dose extrapolation has been used successfully in several wildlife animal species, like gray monkey saki (*Pithecia irrorata*), the kinkajou (*Potos flavus*), the southern two-toed sloth (*Choloepus didactylus*), the pygmy anteater (*Cyclopes didactylus*) and the southern tamandua (*Tamandua tetradactyla*), with reported results ranging from regular to excellent [25]. A comparison of allometric scaling extrapolation with the linear extrapolation of human doses on the use of ketamine in brown howling monkeys (*Alouatta guariba clamitans*) showed superior results by the allometric method, with superior anesthesia time and muscle relaxation [26].

The choice of the model animal is important in allometric scaling extrapolation. Ideally, it should be as taxonomically close as possible to the target animal, and with a similar body mass, as extrapolating from animals too far apart may lead to inadequate results. However, there are no birds among the standard model animals, and the most common domestic animal used as a model is the dog, since the most important trait of the target animal is a very well-defined protocol for the drug used [18,27]. The body mass of model animals is standardized, with the method using a 10 kg body mass for the domestic dog. So, it is important when determining the drug dose for the model animal to use a dose applicable to a 10 kg dog [8].

Allometric scaling extrapolation is based on mathematical models that aim to estimate and predict physiological, anatomical and biochemical parameters between different species and taxa, on the basis that several of these variables exhibit power-law relationship with body size. Through those relationships, pharmacological and physiological variables can be extrapolated from a model animal to a target animal, based on their body mass and taxonomical group [8,27].

Some factors, however, are neglected by allometric scaling and must be considered when designing protocols with this method. Protein binding, toxicity, idiopathic adverse effects and drug sensibility, for example, are independent of body size and related to the species and drug used and, when extrapolating across species, and should be handled outside of allometric scaling [18,27].

Allometric scaling extrapolation cannot claim to, on a short equation, completely adjust anatomical and physiological differences between completely different animals, and this create an error-proof protocol. As veterinary medicine and pharmacology covers hundreds of species of different size and physiology, merging these animals into large and somewhat heterogenic groups becomes a necessity, despite decreasing the accuracy of the method. It does, however, provide a reference point, a guideline to assist the veterinary physician, especially in wildlife medicine [27].

## Conclusions

Chemical restraint in greater rheas with the association tiletamine/zolazepam, in both protocols, provided stability in vital physiological parameters through the entire experimental time and adequate sedation and recovery times, coupled with satisfactory muscle relaxation and immobility. The great similarity between both protocols, despite significant difference in doses, further shows the use of interspecific allometric scaling as an important tool for the veterinary medic in handling wildlife animals.

## Competing interests

The authors declare that they have no competing interests.

## Authors’ contributions

BCP, DASDL and WCL carried out the animal sampling and data collection. MCP, WCL and AMQ contributed to the design of the experiment, provided valuable advice and coordination in the manuscript drafting. BCP, DASDL and MCP performed the statistical analysis and drafted the manuscript. All authors read and approved the final version of this manuscript and are in accordance to its submission.

## References

[B1] Jones TC Hunt RD King NW Patologia Veterinária 2000 6 São Paulo: Manole

[B2] Birdlife International Rhea Americana IUCN Red List of Threatened Speciesversion 2013.1. [http://www.iucnredlist.org]

[B3] Lèche A Della Costa NS Hansen C Navarro JL Marin RH Martella MB Corticosterone stress response of Greater Rhea (Rhea americana) during short-term road transport Poult Sci 2013 92 60 63 10.3382/ps.2012-02377 23243231

[B4] Della Costa NS Lèche A Guzmán DA Navarro JL Marin RH Martella MB Behavioral responses to short-term transport in male and female greater Rheas (Rhea americana) reared in captivity Poult Sci 2013 92 849 857 10.3382/ps.2012-02754 23472006

[B5] Ciboto R Cortopassi SRG Lopes MAE Carvalho RC Baitelo CG Comparison of chemical restraint techniques in ostrich (Struthio camelus) Rev Bras Cienc Avic 2006 8 119 123

[B6] Massone F Anestesiologia Veterinária - Farmacologia e Técnicas 2011 6 Rio de Janeiro: Gunabara Koogan

[B7] Beest JT MacClean M Cushing A Bildfell R Thiafentanil-dexmedetomidine-telazol anaesthesia in greater rheas (Rhea americana) J Wildl Dis 2012 43 802 807 10.1638/2011-0175R2.1 23272347

[B8] Pachaly JR Cubas ZS, Silva JCR, Catão-Dias JL Terapêutica por extrapolação alométrica Tratado de Animais Selvagens – Medicina Veterinária 2006 1 São Paulo: São Paulo: Roca 1215 1223

[B9] Pachaly JR Brito HFV Fowler ME, Cubas PR Interspecific allometric scaling Biology, medicine and surgery of South American wild animals 2001 Ames: Iowa University Press 475 481

[B10] Sedgwick CJ Fowler ME Allometric scaling and emergency care – the importance of body size Zoo and Wild Animal Medicine – Current Therapy 1993 3 Philadelphia, Pennsylvania: W. B. Saunders Co 34 37

[B11] Lin H Tranquilli WJ, Thurmon JC, Grimm KA, Grimm KA Anestésicos dissociativos Lumb & Jones: Veterinary anaesthesia and analgesia 2013 4 Roca: São Paulo 335 384

[B12] Miller RD Anesthesia 2001 5 Philadelphia: Churchill Livingstone

[B13] Hall LW Clarke KW Trim CM Veterinary Anaesthesia 2001 10 Philadelphia, Pennsylvania: W. B. Saunders Co.

[B14] Sumano H Ocampo L Farmacología Veterinária 2006 3 México: México DF

[B15] Medeiros RM Climatologia do município de Teresina 2006 Teresina: Secretaria do Meio Ambiente e Recursos Naturais do Estado do Piauí

[B16] Carvalho HS Ciboto R Baitelo CG Dias RA Cortopassi SRG Anatomia do sistema porta renal e suas implicações no emprego de agentes anestésicos na contenção de avestruzes (Struthio camelus) Cienc Rural 2007 37 1688 1694 10.1590/S0103-84782007000600028

[B17] Ludders JW Matthews NS Tranquilli WJ, Thurmon JC, Grimm KA Lumb & Jones: Veterinary anaesthesia and analgesia 2013 4 Roca: São Paulo 923 952

[B18] Sharma V McNeill JH To scale or not to scale: the principles of dose extrapolation Br J Pharmacol 2009 157 907 921 10.1111/j.1476-5381.2009.00267.x 19508398 PMC2737649

[B19] Lin H Todhunter PG Power TA Use of xylazine, butorphanol, tiletamine-zolazepam and isoflurane for induction and maintenance of anesthesia in ratites J Am Vet Med Assoc 1997 210 244 248 9018361

[B20] Pulgar R Coleccio G Aldana M Pulgar J Estudio comparativo Del efecto de las associaciones anestésicas atropina-tiletamina/zolazepam y atropina-ketamina/diazepam em emúes (Dromaius novaehollandiae) Arc Med Vet 2009 41 149 155

[B21] Belettini ST Alberton LR Silva PRB Stel RF Lourenço WS Pachaly JR Avaliação dos níveis séricos de lactato em cães submetidos a anestesia dissociativa Arq Cien Vet Zool 2008 11 87 95

[B22] Juvenal JC Erdmann RH Moreira N Moraes W Cubas PH Delgado LES Carvalho AL Pachaly JR Contenção farmacológica do gato-do-mato-pequeno, Leopardus tigrinus, para colheita de sêmen, pela associação de tiletamina zolazepam e xilazina Pesq Vet Bras 2008 28 541 546

[B23] Carregaro AB Gerardi PM Honsho DK Allometric scaling of chemical restraint associated with inhalant anesthesia in giant anteaters J Wildl Dis 2009 45 547 551 10.7589/0090-3558-45.2.547 19395770

[B24] Valadão CAA Fantoni DT, Cortopassi SRG Anestésicos dissociativos Anestesia em cães e gatos 2009 2 São Paulo: Roca

[B25] Santana TB Extrapolação alométrica para imobilização farmacológica: avaliação de sua eficácia em sete estudos de caso em silvestres Dissertation 2010 Campinas: Universidade Castelo Branco

[B26] Chagas JAB Oleskovicz N Moraes AN Flôres FN Corrêa AL Souza JC Jr Soares AV Costa A Associação de cetamina S(+) e midazolam pelo método convencional de cálculo e pela extrapolação alométrica em bugios-ruivo (Alouatta guariba clamitans): resposta clínica e cardiorrespiratória Cienc Rural 2010 40 109 114 10.1590/S0103-84782010000100018

[B27] Mahmood I Application of allometric principles for the prediction of pharmacokinetics in human and veterinary drug development Adv Drug Deliver Rev 2007 59 1177 1192 10.1016/j.addr.2007.05.015 17826864

